# Segregated neural explants exhibit co-oriented, asymmetric, neurite outgrowth

**DOI:** 10.1371/journal.pone.0216263

**Published:** 2019-09-05

**Authors:** David B. Pettigrew, Curtis B. Dobson, Lori G. Isaacson, Eric C. Leuthardt, Heather N. Lilley, Georgette L. Suidan, Keith A. Crutcher

**Affiliations:** 1 Department of Anatomy and Neuroscience, Department of Physical Therapy, University of Findlay, Findlay, Ohio, United States of America; 2 Medical Device Biology Group, Division of Pharmacy and Optometry, The University of Manchester, Manchester, England, United Kingdom; 3 Department of Biology, Miami University, Oxford, Ohio, United States of America; 4 Department of Neurosurgery, University of Cincinnati College of Medicine, Cincinnati, Ohio, United States of America; Boston Children’s Hospital and Harvard Medical School, UNITED STATES

## Abstract

Explants of embryonic chick sympathetic and sensory ganglia were found to exhibit asymmetric radial outgrowth of neurites under standard culture conditions with or without exogenous Nerve Growth Factor [NGF]. Opposing sides of an explant exhibited: a) differences in neurite length and, b) differences in neurite morphology. Strikingly, this asymmetry exhibited co-orientation among segregated, neighboring explants. The underlying mechanism(s) of the asymmetry and its co-orientation are not known but appear to depend on cell clustering because dissociated sympathetic neurons do not exhibit co-orientation whereas re-aggregated clusters of cells do. This emergent behavior may be similar to the community effect described in other cell types. If a similar phenomenon exists in the embryo, or in maturity, it may contribute to the establishment of proper orientation of neurite outgrowth during development and/or injury-induced neuronal plasticity.

## Introduction

Neurons exhibit highly differentiated phenotypes and are among the most morphologically diverse cell types known. Most notable are the cellular extensions [neurites], which develop into dendrites and axons and can extend several feet in some organisms. In mammals, most neurons are also highly polarized, with the dendritic tree usually occupying a position opposite to that of the axon. When neurons are dissociated, placed into tissue culture, and given an appropriate substrate, they also extend neurites that, in some cases, mimic their phenotype in vivo, e.g., hippocampal axons and dendrites [1]. Neurons can also be cultured as tissue explants and, under permissive conditions, will exhibit a profusion of neurites that form a radial halo around the core of the explanted tissue [2].

This explant assay, initially using chick sensory or sympathetic ganglia, provided a means of detecting neurite growth-promoting substances such as Nerve Growth Factor [NGF] [2] and remains a useful bioassay to identify factors that stimulate or inhibit neurite growth in vitro. We used this explant assay over several years to detect and quantify neurite outgrowth in a variety of experimental situations [3–7]. The method, established by the early experiments of Levi-Montalcini and co-workers, involves dissection of embryonic chick sympathetic or sensory ganglia and placing small pieces [approximately 1 mm^2^] of the tissue in culture dishes coated with a suitably adhesive substrate [typically poly-ornithine] that permits attachment of the explants as well as extension of neurites. When grown in a suitable culture medium, the result is a radial halo of neurites [8–10].

In early experiments we observed that the neurite halo often had longer neurites on one side of the explant despite the absence of any known tropic signals. Moreover, the halo was often asymmetric with differences in neurite morphology such that one side often exhibited a dense halo, often with flattened club-like neurites and, on the opposite side, a sparser halo with thinner neurites. Asymmetry in both neurite length and morphology occurred under standard culture conditions with or without exogenous NGF. Examples of asymmetric neurite outgrowth have been documented in the literature but without comment [see discussion].

The presence of asymmetric neurite outgrowth allowed for an even more unexpected observation. The halos from adjacent, but separate, explants often exhibited co-orientation of their halos, with the longest neurites extending in the same direction. Such co-orientation was not exhibited by dissociated neurons under the same culture conditions, but was present when dissociated neurons were re-aggregated, suggesting that this is an emergent property of cell aggregates.

## Materials and methods

Sympathetic chain ganglia or sensory ganglia were dissected from embryonic chickens ranging in age from E9 to E11. Following removal from the embryo, the ganglia were cut into small explants [approximately 1 mm^3^ in size] and placed in 35 mm diameter dishes [Falcon 1008, Fisher Scientific, Houston, TX], or 6 or 12-well, multi-well dishes, that had previously been coated overnight with poly-l-ornithine in borate buffer [pH 8.35]. Various culture media have been used but most cultures were established either in Ham’s F12 or in serum-free Neurobasal Medium with B27 supplement [Life Technologies, Gaithersburg, MD] [11] with or without added NGF or proNGF in solution. Culture durations ranged from 2–4 days. Following culture, in order to visualize the neurite halo, the explants were either fixed and stained for 15 minutes with a 20% silver nitrate solution for 15 minutes followed by a 10% silver nitrate solution for 5–30 minutes, or the living cultures were loaded with a vital dye, 15 ng/ml 5-carboxy-fluorescein diacetate AM [Molecular Probes, Eugene, OR], for 45–90 min at 37°C in Ham’s F12 medium [Sigma, St. Louis, MO].

For other experiments, sympathetic neurons were dissociated by enzymatic digestion to assess outgrowth of individual neurons. Sympathetic chain ganglia were incubated with 0.25% trypsin [Sigma] for 20 min at 37°C. Trypsinization was subsequently blocked by exposure to 100% heat-inactivated fetal bovine serum [Harlan Bioproducts, Indianapolis, IN] for five minutes, and washed three times with serum-free Ham’s F12 medium [Sigma, St. Louis, MO]. The tissue was then dissociated by gentle trituration using flamed Pasteur pipettes [Fisher Scientific, Houston, TX]. In one experiment, the dissociated cells were then allowed to reaggregate before placing them in culture to assess whether dissociation would interfere with the expression of explant asymmetry and co-oriented outgrowth.

A typical explant gave rise to neurites that extended radially to form a “halo.” The resulting halo could be circular or elliptical, i.e., with a short minor axis and a longer major axis. The explant core [containing neuronal cell bodies] may occupy a position in the center of the halo or offset along the major axis [but not along the minor axis], giving rise to an asymmetric halo, albeit with bilateral symmetry on either side of the major axis. In other words, neurites systematically varied in length at different positions around the explant. In order to quantify the extent of neurite length asymmetry, a macro was developed for NIH ImageJ [source code is available upon request]. The perimeters of the halo and explant core were outlined and recorded by the macro. The macro fit an ellipse to the halo and identified its major axis. The macro determined the magnitude of neurite length asymmetry by measuring the distance along the major axis from the midpoint of the major axis, i.e., the center of the halo, to the explant core center. The magnitude of the neurite length asymmetry was calculated as the percent distance of the core center from the halo center to the closer end of the major axis. Thus, it was theoretically possible to have a maximum asymmetry of 100% if the entire halo extended from only one side of the explant, a phenomenon that was never observed in our sample. In contrast, a symmetric halo, such that the explant core is directly at the midpoint of the major axis, would generate an asymmetry value of 0%.

In some cultures, there was asymmetry in neurite morphology with little visible asymmetry in neurite length. This was especially the case in silver-stained explants where the longest individual neurites were not always visible at low magnification. In order to compare the orientation of such explants based on the morphological asymmetry, images were analyzed by blinded observers. Images of individual explants were created using round image fields to eliminate cues caused by square edges. The images were then rotated by a random angle [each explant was rotated by a different angle]. Two observers, blinded to the original orientation, were asked to draw an equatorial line that best demarcated the morphologically distinct sides of the halo. The observers were also asked to indicate which side of the equator corresponded to the sparser halo with thinner neurites [see description in results]. The data were decoded by reversing the random rotation. The axis of orientation of the halo was taken to be the angle of the line perpendicular to the equatorial line, in the direction of the sparser halo with thinner neurites. The angles measured by the two observers were averaged and plotted as axes on a polar plot. The hypothesis that the explants were co-oriented was tested using the Rayleigh test [12]. Axes of orientation were compared and p-values were computed based on the von Mises distribution [circular normal distribution].

The outgrowth from dissociated neurons consists of a plexus of many intersecting neurites. To assess whether there was a prevailing direction of growth from these cultures, Fourier transforms of representative fields were captured. The Fourier transform shows a polar plot of spatial frequency content. In the case where neurites are organized in parallel with each other, the Fourier Transform detects high frequency content in the direction perpendicular to the neurites and low frequency content in the direction parallel to the neurites. Consequently, organized parallel growth produces an elongated perpendicular band of high signal in the Fourier transform. Unorganized growth, in contrast, produces shorter, equal-length bands of high signal at all angles. This method was used to determine if there is underlying co-orientation of outgrowth from dissociated neurons with co-cultured explants.

## Results

**Fig 1:**
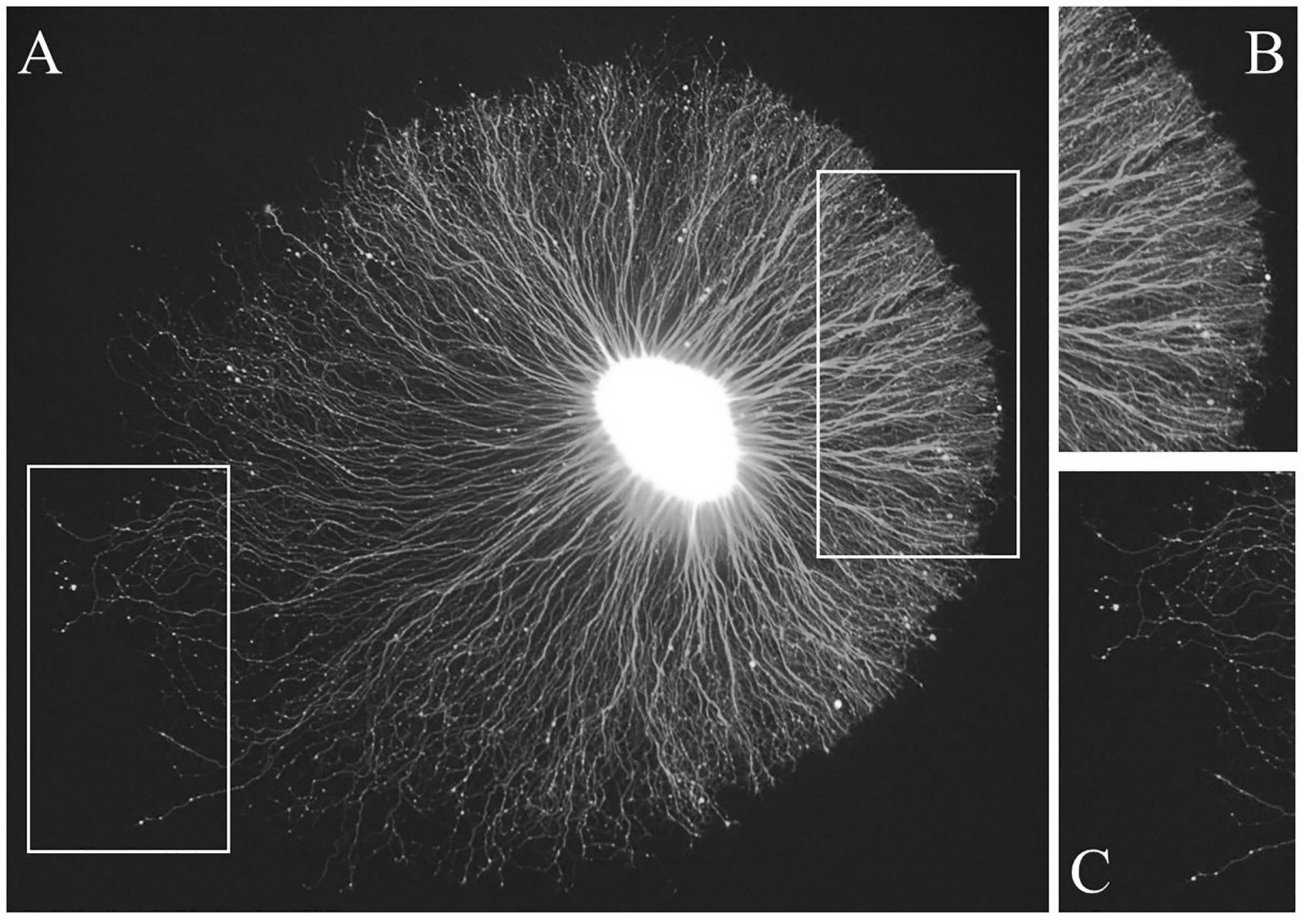
An example of neurite outgrowth from an embryonic chick sympathetic ganglion explant cultured in the presence of Nerve Growth Factor [NGF] stained with vital dye. The tissue has been labeled with a vital dye to reveal the core [bright central area] and surrounding neurites [A]. The neurite halo shows asymmetric outgrowth with the shorter neurites extending to the right side where they form a well-defined edge [inset shown in panel B]. In contrast, the neurites on the left side are thinner and show more variable lengths [inset shown in panel C].

A typical example of a sympathetic explant with an extensive neurite halo is shown in Fig 1. In this case, the living explant has been labeled with a vital dye. The brightly stained spherical core consists of neuronal cell bodies, the initial segments of their neurites, and Schwann cells. In this example, the neurite halo is asymmetric, i.e., it is wider from the lower left to the upper right of the image [major axis] than from the upper left to the lower right [minor axis]. Along the major axis, the explant core is closer to the right side of the halo. In other words, the neurites are longer on one side of the halo than the other. Furthermore, on the right side, the neurites are densely packed and exhibit a distinct boundary in contrast to those on the left side where the neurites appear thinner and the boundary is less distinct.

**Fig 2:**
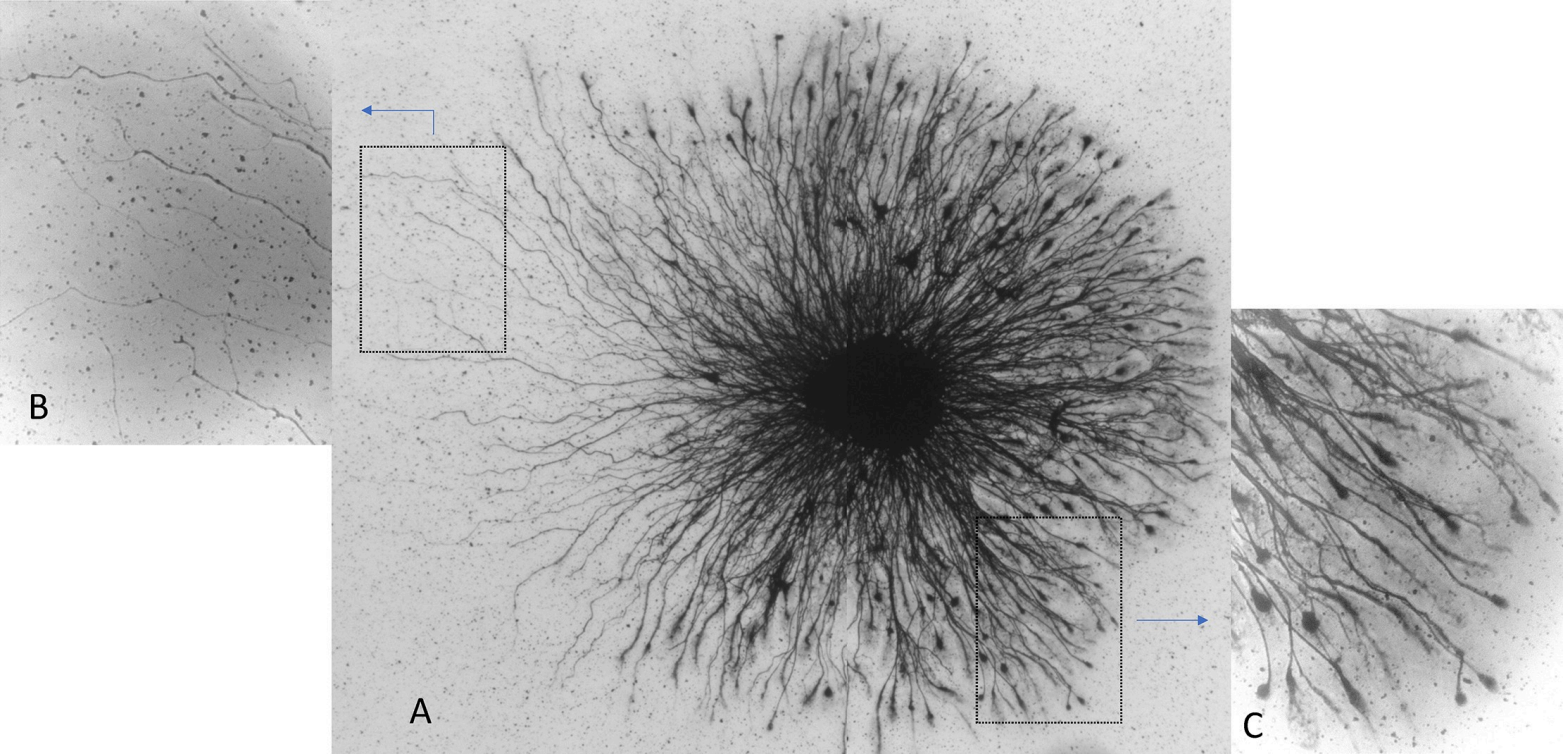
An example of neurite outgrowth from an embryonic chick sympathetic ganglion explant stained with silver. The neurite halo shows asymmetric neurite growth [A] such that the neurites on the left side are longer and thinner [B] than those on the right side, which also have distinct club-like endings [C].

An additional example of asymmetric outgrowth is shown of a sympathetic explant that has been stained with silver [Fig 2A]. As in the example shown in Fig 1, the halo is asymmetric with longer neurites on one [the left] side and there is a clear morphological difference between the two sides of the halo perimeter. On the left side [Fig 2B], the neurites are separated and variable in length whereas on the right side [Fig 2C] the neurites are densely clustered, and many have flattened, club-like endings that are absent on the left. In general, the difference in neurite length is easier to discern with the vital dye-stained explants and the morphological differences are best visualized with the silver stain.

Although the examples shown in Figs 1 and 2 are representative of the types of asymmetric outgrowth observed with both vital dye and silver staining, there was variability in the appearance of the halos across different cultures. For example, not all explants exhibited asymmetry, either in neurite length or morphology. Furthermore, if explants were located close to the edge of the dish it was not possible to determine whether a halo was asymmetric.

**Fig 3:**
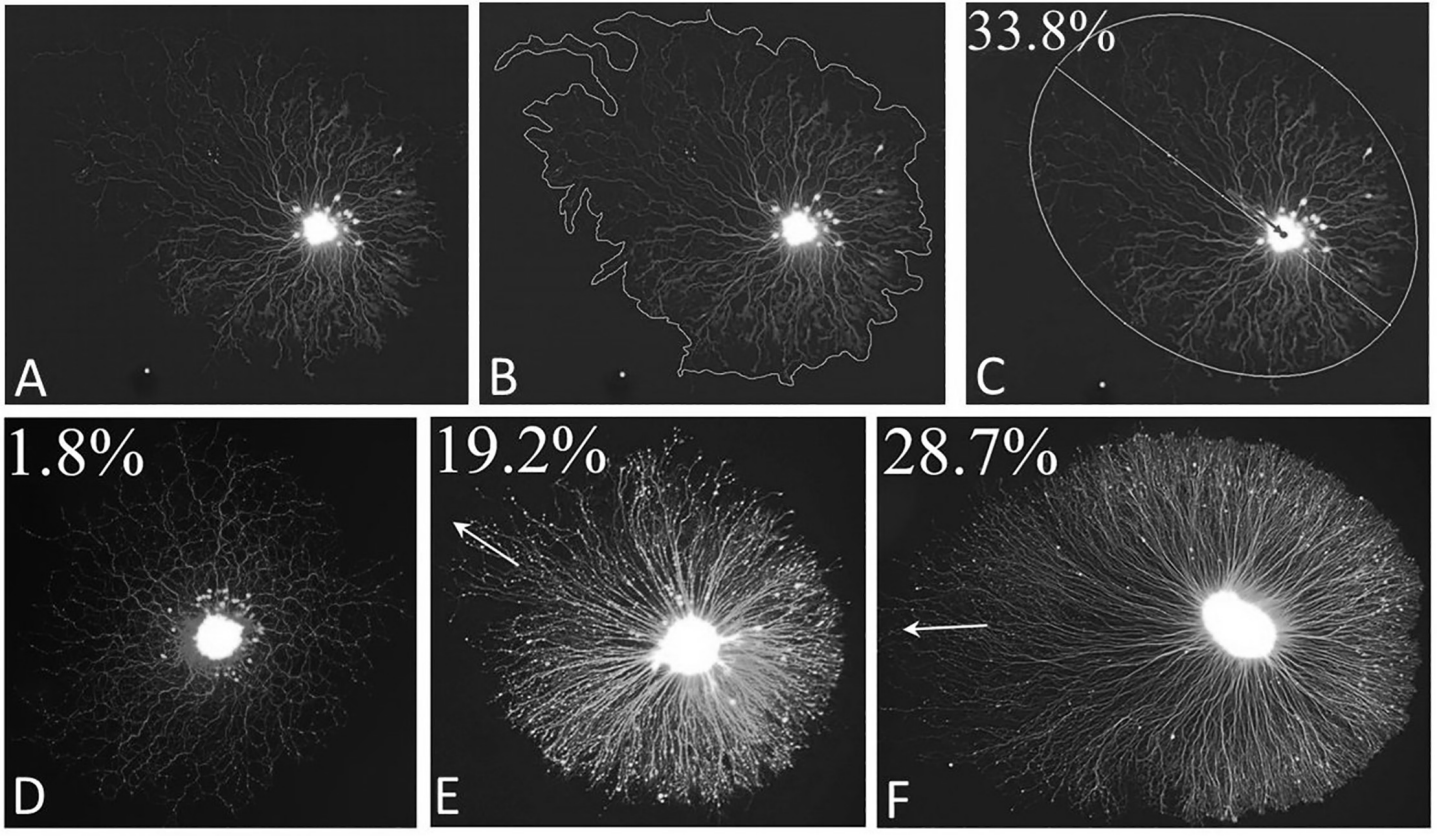
Quantification of the degree of neurite length asymmetry and its orientation. An example of the method is shown using the explant in panel A. A contour was drawn around the explant halo [B] and explant core [not shown] using the ImageJ Freehand selection tool. Using the “Fit Ellipse” tool, ellipses were fit to the halo [C] and the core [not shown]. The centers and major axis lengths of both ellipses were identified by the macro. The center of the explant core was compared to the distance from the halo center to the nearest end of the major axis [arrow in C]. The magnitude of neurite length asymmetry was taken to be the percent displacement of the core center from the halo center to the nearest end of the major axis [in this case, 33.8%]. Examples of other explants analyzed in this way are shown in D-F.

Although we also observed asymmetric outgrowth with explants of chick sensory ganglia [see below], the majority of our cultures were established with sympathetic explants. Therefore, we analyzed the prevalence and extent of outgrowth asymmetry with this tissue. Neurite length asymmetry was measured using a customized NIH Image J macro to define the relationship between the explant core and its corresponding halo as described above [Fig 3A, 3B and 3C]. The halo perimeter exhibits an irregular border, especially in the region where neurite length is most variable. Neurite length asymmetry was expressed as the percent distance of the center of the explant core from the center of the major axis of the halo. In the example shown [Fig 3A, 3B and 3C] asymmetry was estimated at 33.8%, i.e., the explant core is displaced 33.8% towards the lower right side of the halo. Examples of the extent of neurite length asymmetry calculated in this manner are shown in Fig 3D, 3E and 3F. The extent of asymmetry measured in this way showed examples with virtually no neurite length asymmetry [Fig 3D, 1.8%], where the explant halo is virtually symmetric, and other cases in which there is greater asymmetry [Fig 3E, 19.2%] and [Fig 3F, 28.7%].

**Fig 4:**
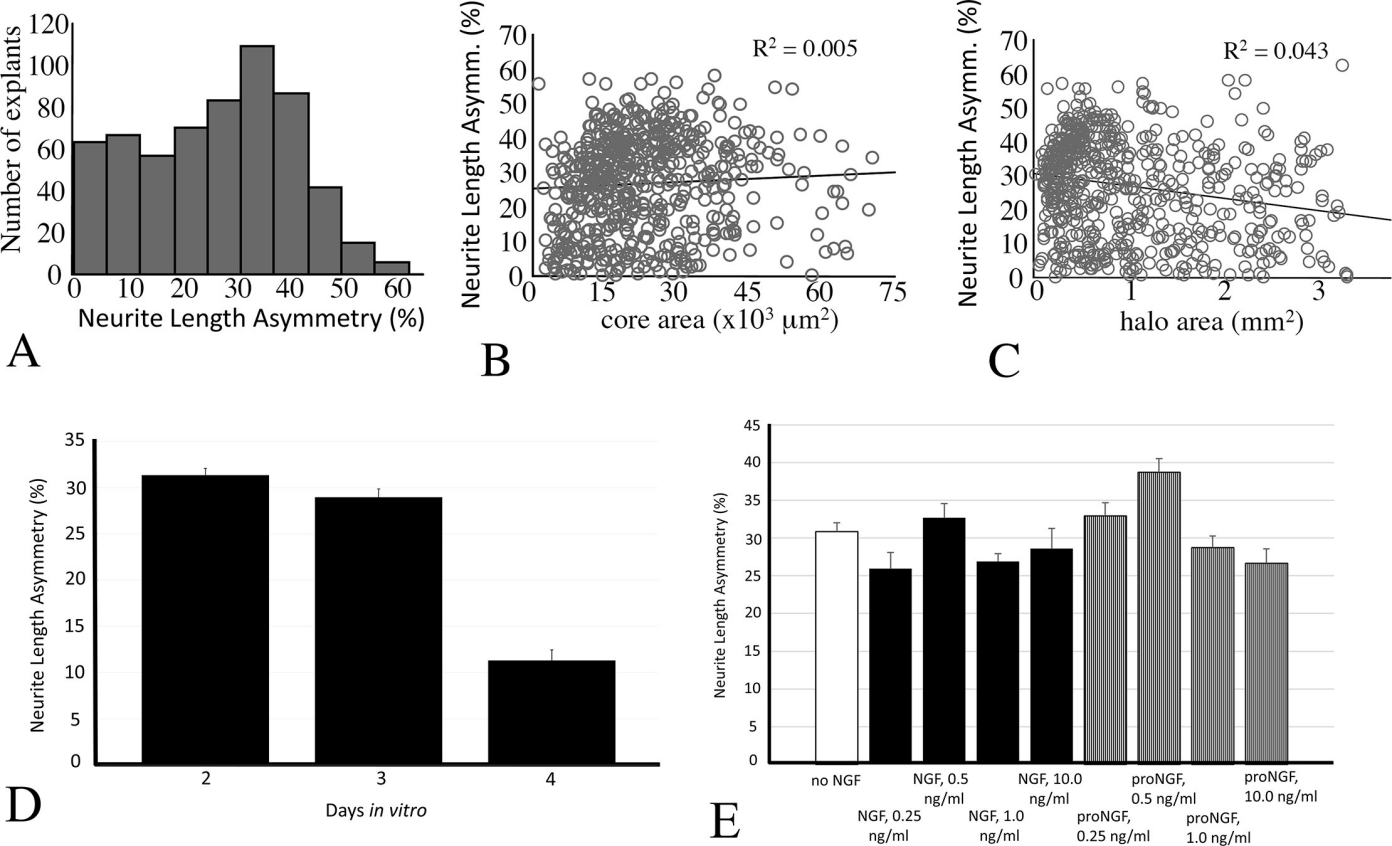
Prevalence and extent of neurite length asymmetry. [A] Histogram showing the prevalence of neurite length asymmetry in 603 explants cultured in Neurobasal medium for 2–4 days and stained with vital dye. The majority showed asymmetry of at least 15%. Bivariate scattergrams showing the distribution of asymmetry as a function of explant core [B] and halo [C] areas. The extent of neurite length asymmetry showed no direct correlation with either variable. Neurite length asymmetry diminished by day 4 [D]. Neurite length asymmetry was present without treatment with NGF, and treatment with NGF or proNGF at various concentrations [E]. Greatest neurite length asymmetry was observed with 0.5 ng/ml NGF or proNGF. Bars in [D] and [E] represent mean ± SEM.

To obtain some indication of the prevalence and extent of neurite length asymmetry, 603 explants from thirteen cultures grown in Neurobasal Medium for 2–4 days and then stained with vital dye were analyzed according to the procedure described above. As shown in the histogram in Fig 4A, explants showed neurite length asymmetry ranging from 0 to 60% with the mode of the distribution around 35%. Of these 603 explants, 440 of the explants [73%] exhibited an asymmetry of at least 15%. The extent of neurite length asymmetry did not correlate with either core area [Fig 4B] or halo area [Fig 4C]. Average neurite length asymmetry varied as a function of culture duration [ANOVA, *p* < 0.05], decreasing substantially by day 4 [Fig 4D]. The explants were treated with various concentrations of NGF or its precursor, proNGF [Fig 4E]. While neurite length asymmetry was observed with all treatments, there were slight, but statistically significant, effects of both NGF form [*p* < 0.02] and concentration [*p* < 0.0001] with 0.5 ng/ml of either NGF or proNGF producing the greatest neurite length asymmetry [two-way ANOVA]. There was no significant interaction between NGF form and concentration [*p* = 0.18].

**Fig 5:**
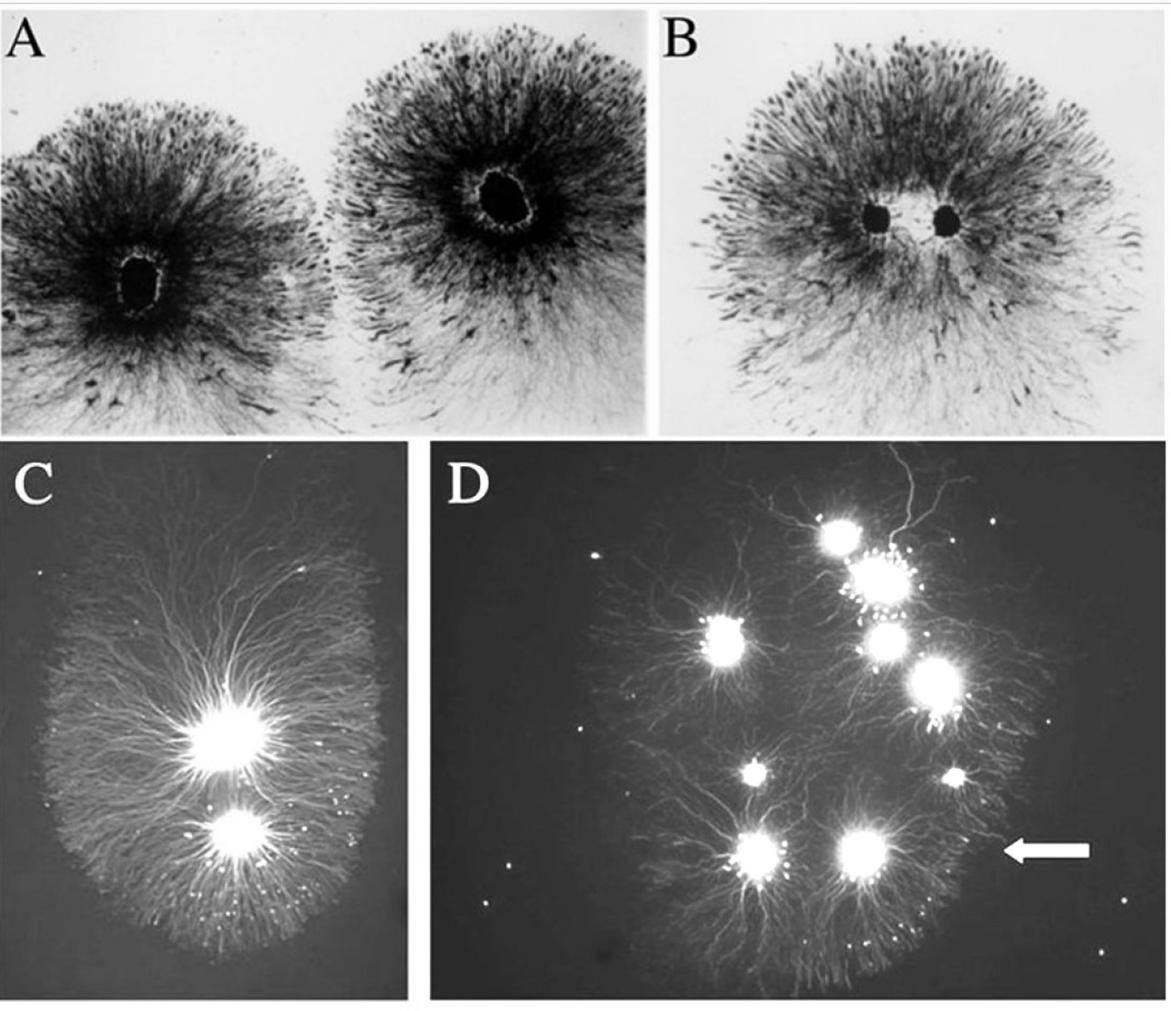
Explants with shared halos. Two explants growing close to each other have extended asymmetric halos, each of which shows similar orientation [A]. Two explants may contribute to a common halo that shows asymmetry with the cores situated side-by-side [B] or in “tandem” [C]. A collection of nine explants has extended halos that collectively exhibit a front of organized neurites [arrow] on one side that are shorter than those on the opposite side of the collective halo [D].

In addition to the common occurrence of outgrowth asymmetry, another phenomenon was observed when multiple explants were cultured in the same dish, whether visualized with vital dye or silver stain. In cases where two asymmetric explants were adjacent to each other but without physical contact, they usually shared a common orientation [Fig 5A]. Two or more explants in sufficient proximity to each other would also extend a common asymmetric halo [Fig 5B, 5C and 5D]. For the analysis of both asymmetry and co-orientation, only explants that were located away from the dish edge and physically separated from each other were included [see below].

**Fig 6:**
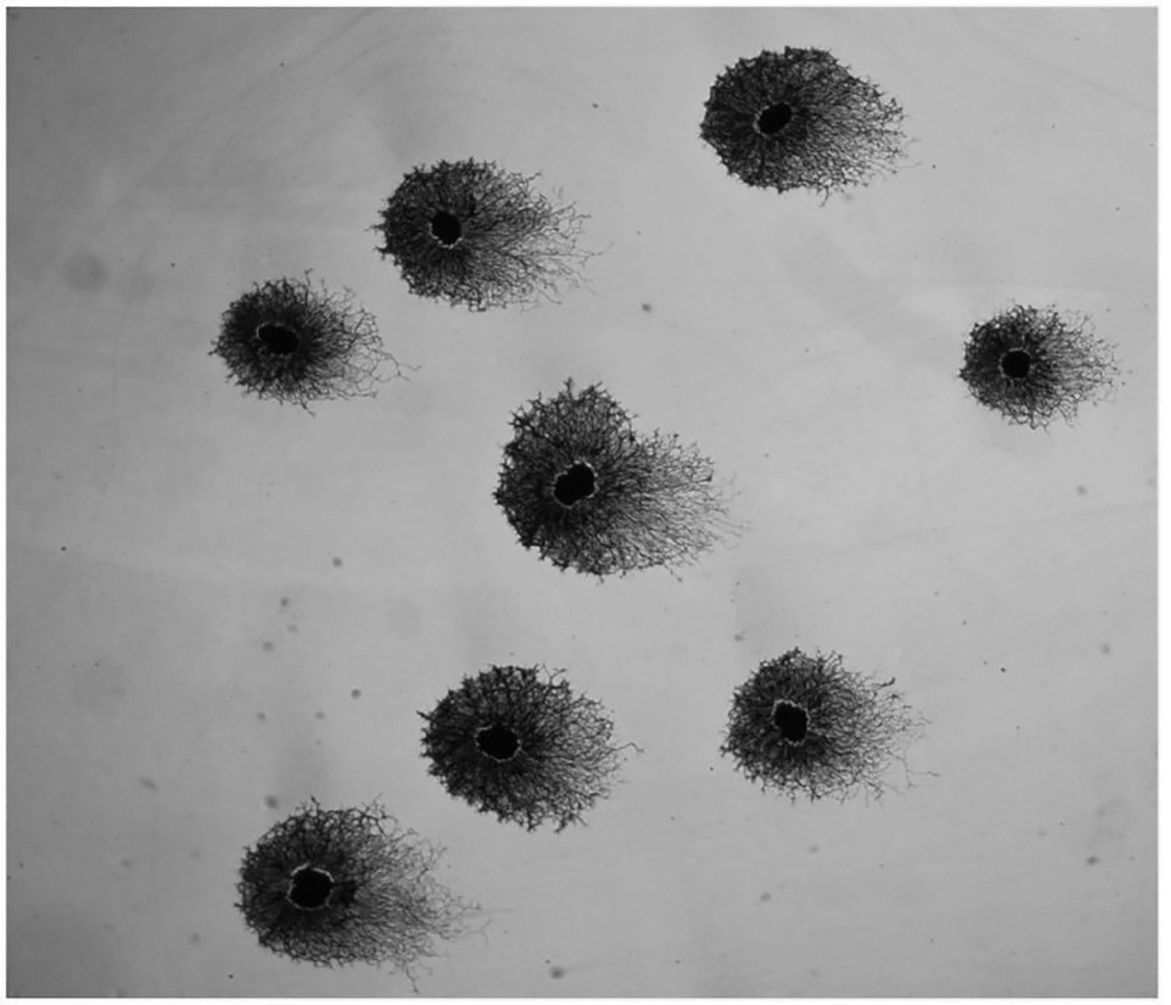
A field of silver-stained sympathetic explants exhibiting co-orientation of neurite halos. Each of the eight explants exhibits an asymmetric halo and the longest neurites within each halo extend to the right side of the field. Such co-orientation of halos was a common occurrence among neighboring explants although there was no consistent orientation from one culture dish to another.

In cases where explants were segregated, their asymmetric halos exhibited co-orientation with neighboring explants. This was best observed with silver staining because of the ability to visualize several explants with low power magnification using bright field illumination. An example of a field of eight segregated sympathetic explants in a single dish is shown in Fig 6. Each shows evidence of asymmetric neurite outgrowth, in neurite length, density and morphology. In addition, the orientation of the longest neurites in each halo is in the same direction, in this case toward the right side of the field. None of the explants are in physical contact with each other. Furthermore, co-orientation was usually a local phenomenon, i.e., explants on opposite sides of the dish, did not necessarily have the same orientation. In experiments involving several dishes incubated at the same time, there was no indication that the explants in one dish were oriented in the same direction as explants in an adjacent dish.

**Fig 7:**
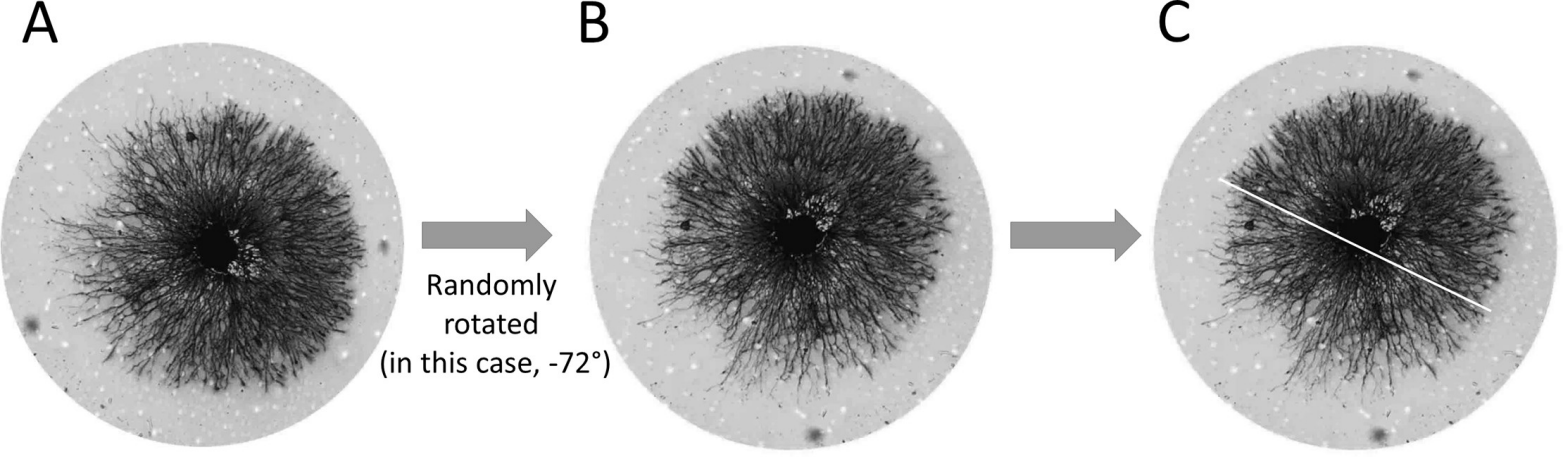
Method for quantifying the direction of morphological asymmetry. Each explant in a group was cropped using a circular field [A]. The image of each explant was rotated randomly. A different random rotation factor was used for each explant. In this example, the explant was rotated counterclockwise by 72 degrees [B]. An observer blinded to the original orientation was asked to assess the orientation of the rotated halo based on growth cone morphology [C]. The observer was asked to draw a line that best demarcates morphological differences on opposite sides of the halo.

In some instances, there was morphologic asymmetry but limited or no neurite length asymmetry. This was especially the case with explants stained with silver because the longest individual neurites were not always visible at low magnification. To quantify the orientation of halos showing morphological asymmetry, images of individual explants were cropped out using round fields to eliminate extrinsic cues created by square edges and each rotated by a different random angle [Fig 7]. Blinded observers were then asked to draw an equatorial line that best separated the morphologically distinct sides of the halos and indicate which side of the halo showed the sparser halo with thinner neurites. The axis of orientation of the halo was defined as the angle perpendicular to the equatorial line, in the direction of the sparser halo with thinner neurites, after reversing the random rotation.

**Fig 8:**
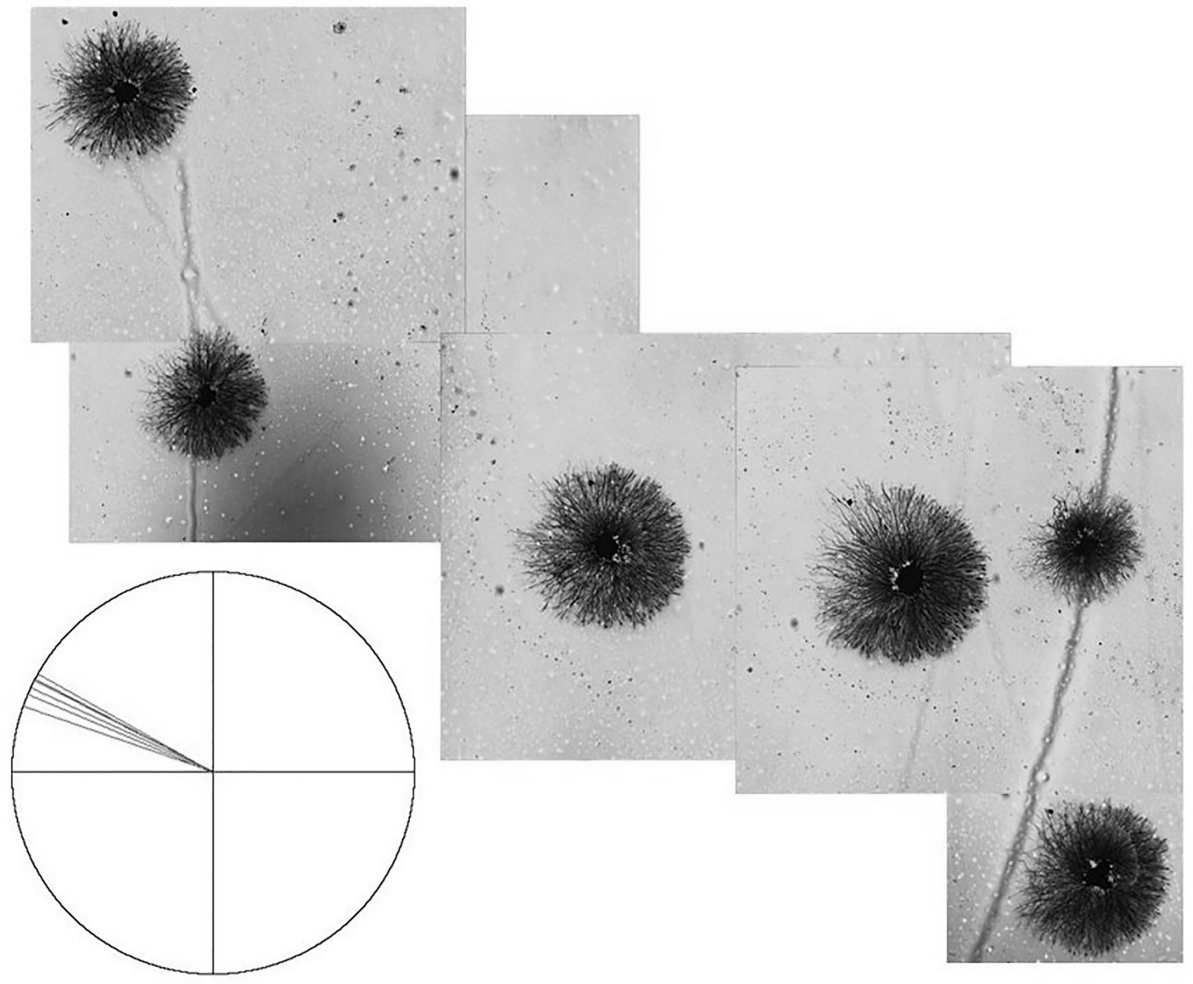
Six silver stained explants with cores centered within circular neurite halos. Morphologic differences in the halo are evident, with tightly packed, well organized fronts of neurites to the right and less densely packed, less organized fronts of neurites to the left. The polar diagram [inset] plots the average axis of orientation for each explant, showing they are highly co-oriented based on blinded assessments of asymmetry [Rayleigh r = 0.998; p < 0.004].

Another example of a culture in which several explants show co-orientation is shown in Fig 8. In this example, there is little asymmetry in neurite length [although the silver stain does not reveal the longest individual neurites at this magnification] but there does appear to be asymmetry in neurite morphology. In order to evaluate the extent of co-orientation, the six explants in this field were evaluated using the previously described method by two subjects blinded to the original orientation. Axes of orientation for each explant were calculated based on the equatorial lines drawn by the two observers and the two values were averaged for each explant. The averaged axes of orientation were plotted on the polar diagram in Fig 8 [inset, each line indicates the orientation of one explant halo]. If the orientation of the explants was random, the lines would be distributed around the circle. In fact, all the lines are directed toward the upper left and clustered in the upper left quadrant of the circle, indicating a non-random distribution. Rayleigh analysis of the polar plot of these averaged axes demonstrated statistically significant co-orientation [r = 0.998; *p* < 0.004].

**Fig 9:**
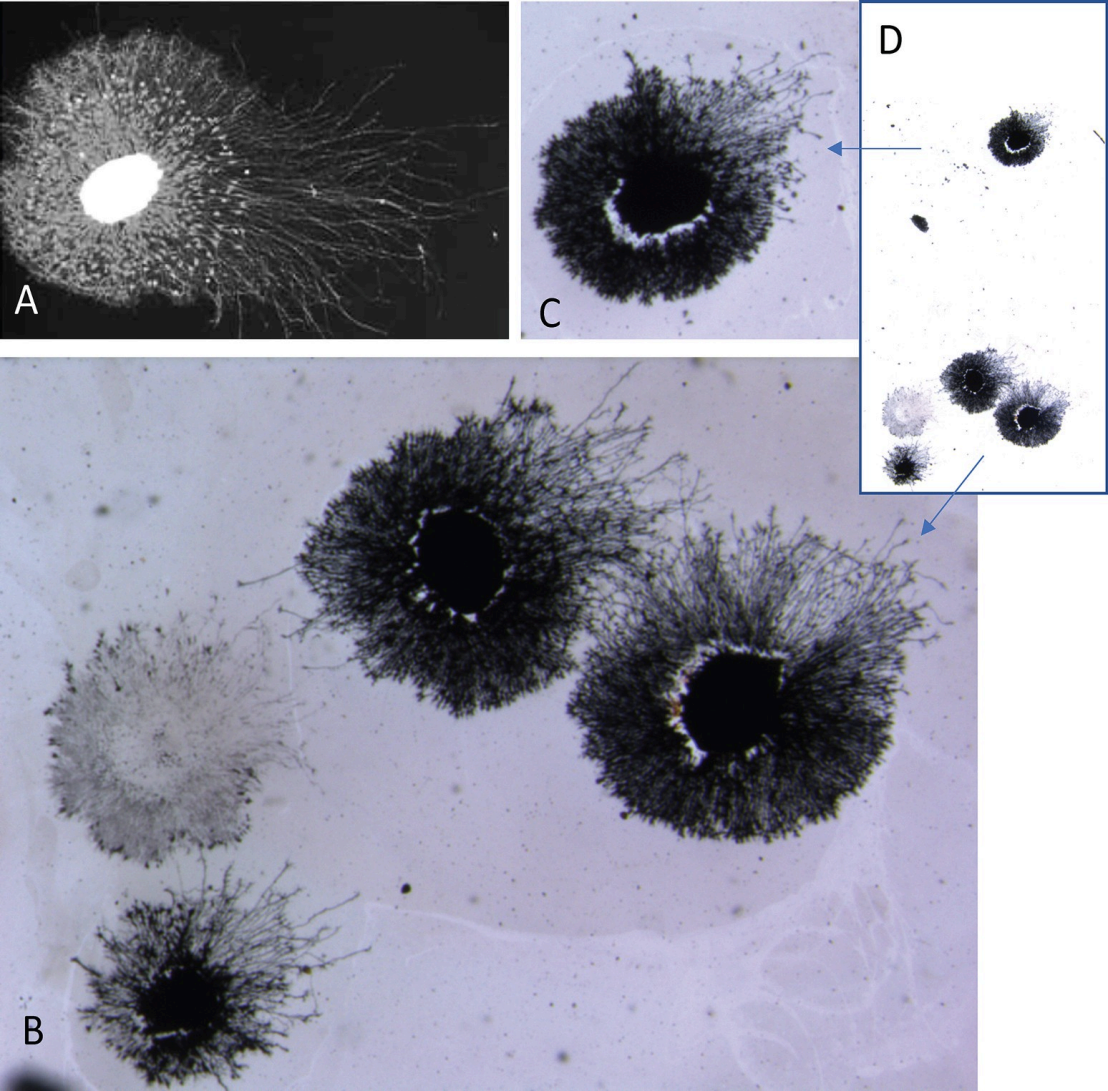
Sensory ganglia explants exhibit asymmetry and co-orientation. An example of a sensory ganglion explant stained with vital dye exhibiting asymmetric neurite growth [A]. A cluster of four silver-stained sensory explants show co-oriented asymmetry [B] along with a more distant explant in the same culture dish [C]. The inset [D] shows the distribution of the five explants in the dish. The upper left explant core in the cluster was lost during the staining procedure but the remnants of the neurite halo remain.

The vast majority of our cultures used embryonic chick sympathetic ganglia. However, we have also observed asymmetric growth in sensory explants from chick embryos of comparable age [Fig 9]. The asymmetric outgrowth is visible with both vital dye [Fig 9A] and silver staining [Fig 9B, 9C and 9D]. As was the case for the sympathetic explants, there is a clear distinction between the opposite sides of the explant such that the neurites on one side are longer. One difference between the sympathetic and sensory explants visualized with vital dye is that the sensory explants have an extensive halo of non-neuronal cells that almost reaches to the limit of the shorter neurite border [Fig 9A]. However, unlike the neurite outgrowth, the distribution of these non-neuronal cells is not notably asymmetric.

Ten cultures of sensory explants with exogenous NGF were established to determine whether co-oriented asymmetry also occurs with this neural tissue. Fig 9B, 9C and 9D shows an example of one such culture stained with silver in which a cluster of four sensory explants exhibit co-oriented asymmetry [Fig 9B]. The longest neurites are all oriented in the same general direction for each explant. A fifth explant [Fig 9C] at some distance away from the cluster [Fig 9D] also exhibits asymmetric growth with an orientation similar to that of the cluster. Of the 33 explants with neurite outgrowth from all ten cultures, 4 were located near the edge of the dish such that it was impossible to determine whether outgrowth was asymmetric. Of the remaining 29 explants, 22 [75%] showed asymmetric growth. Six of the cultures had two or more asymmetric explants sufficiently separated from each other and the edge of the dish to detect co-orientation. Three of these cultures showed clear co-oriented asymmetry, as shown in Fig 9. Co-orientation was not obvious in the other three cultures where the explants were more separated from each other.

**Fig 10:**
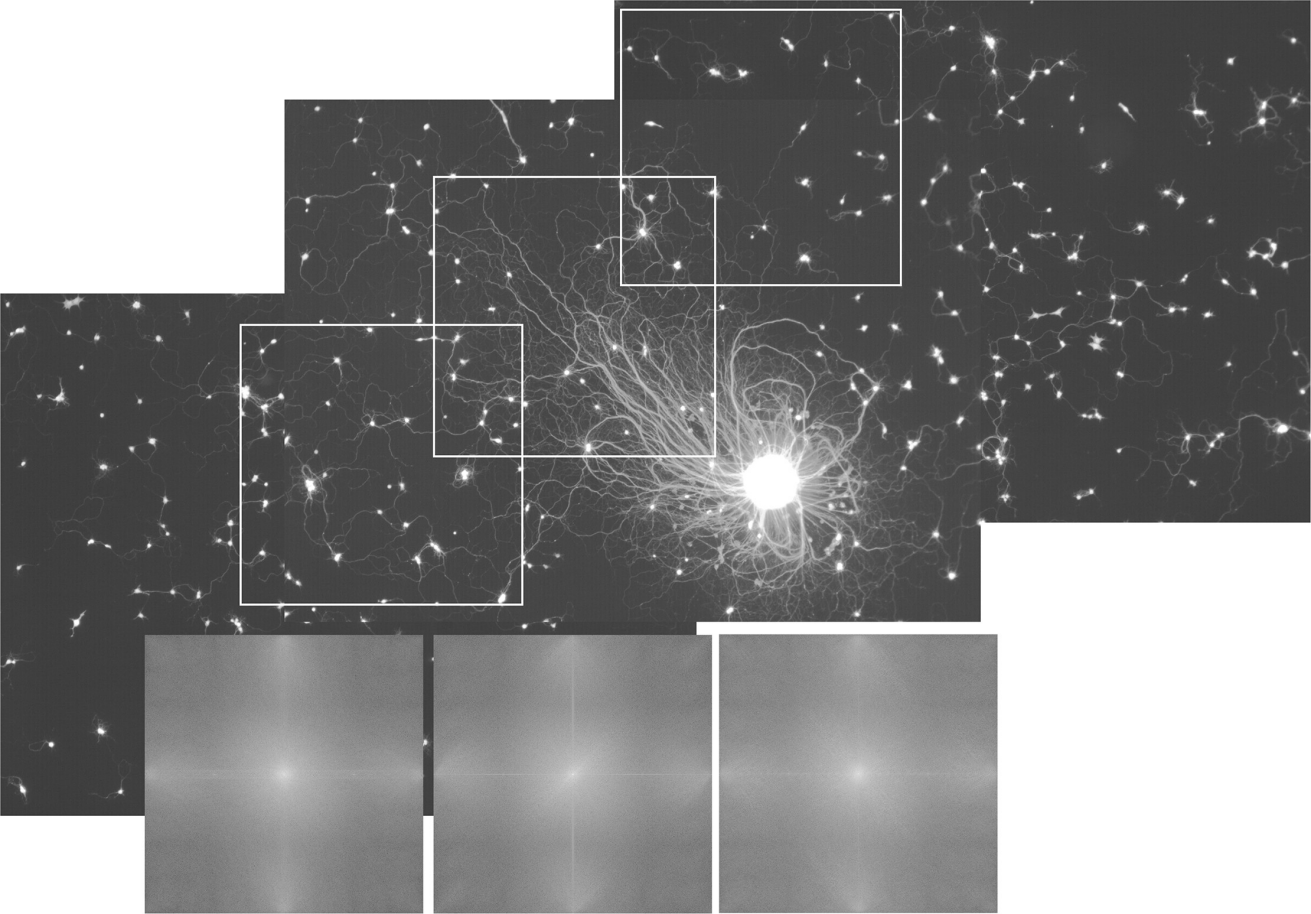
A sympathetic explant with asymmetric outgrowth. The longest neurites of the explant extend towards the upper left of the field contained in the middle box. This explant was co-cultured with dissociated neurons, which show no consistent orientation in outgrowth. The insets show Fourier transforms of the three respective white-bordered subfields showing that only the explant halo shows outgrowth in predominantly one direction [indicated by the bright line extending from the lower left to the upper right quadrant in the middle transform].

To determine whether the orientation of explant halos was also reflected in the orientation of neurites from individual cells in the same cultures, we combined dissociated sympathetic cells with sympathetic explant cultures [Fig 10]. In spite of the clear asymmetry in the neurite halo of the explant, there was no apparent directional orientation of outgrowth from individual neurons. This conclusion is supported by Fourier transforms captured of the long side of the explant halo and two adjacent fields containing only dissociated neurons. The middle Fourier transform corresponds to the portion of the explant halo contained within the middle box with the white border. An elongated perpendicular band of high signal can be observed in the transform oriented perpendicular to the predominant direction of neurites within the halo, i.e., extending from lower-left to upper-right. The left-most and right-most Fourier transforms correspond to the left-most and right-most fields of dissociated neurons, respectively. No band similar to that in the middle transform is present.

**Fig 11:**
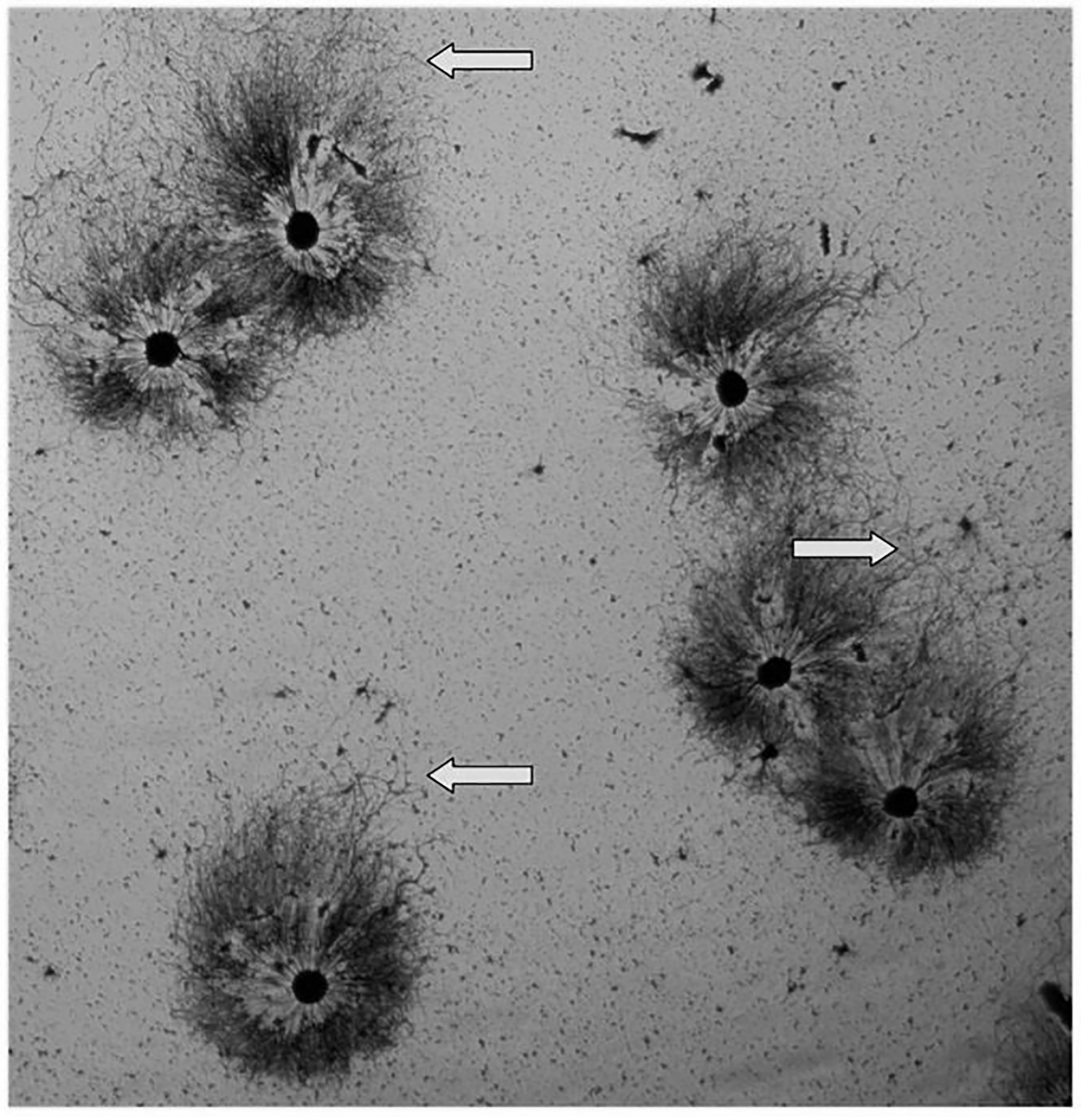
A field of “reaggregated” explants is shown stained with silver nitrate. The ganglia were first dissociated, and the cells were then allowed to form clusters before plating. The resulting neurite halos exhibit asymmetry and the longest neurites extend in the same general direction [towards the top of the field, as indicated by the arrows]. The neurites on the opposite side of each halo tend to be shorter and to establish a sharper boundary.

Finally, to assess the possibility that the asymmetry and co-orientation depend on cues retained ex vivo after being established in the embryo, we dissociated sympathetic ganglia and then allowed the cells to re-aggregate before placing them in culture. The re-aggregated explants also showed asymmetric co-oriented outgrowth such that the longest neurites extended in the same direction [arrows in Fig 11].

## Discussion

This study demonstrates a seemingly simple phenomenon whereby explants of embryonic chick sympathetic or sensory ganglia exhibit asymmetric neurite outgrowth in nominally uniform growth environments. Furthermore, this asymmetric growth shows local co-orientation and is observed with cell aggregates but not with single cells. Whether this emergent asymmetry depends on the number of cells and/or the presence of non-neural cells in the explants is unknown. In either case, the results are consistent with the speculation for a tissue-level mechanism that may operate during development and/or may have played a role in the evolutionary transition to multicellularity.

The neurite halo assay was first developed to detect neurite growth-promoting substances released by tumor tissue [2,13,14] and has been used by numerous investigators. The original description of the results obtained when neural tissue was co-cultured with tumor tissue is similar to our results. The neurites from a sensory explant facing the tumor were described as showing “*maximal density and a very straight course*” whereas those on the opposite side were described as “*less dense and longer; the direction of their outgrowth is less straight*, *and some take more winding routes*” [2]. The majority of explants shown in most of the early publications using this method do not include the full explant halo, so it is difficult to appreciate the asymmetry being described. However, a number of images from this original work can be found, including one on the cover of a book published by Levi-Montalcini clearly showing asymmetry [15]. The same example was used in a review paper [16]. In fact, the description of halo asymmetry in the 1954 paper cited above could well apply to the example shown in our Fig 1. The main experimental difference is that our neural explants were never cultured in the presence of non-neural tissue. Nor did we observe explant halos oriented towards each other.

The shape of the neuritic halo from “control” explants was described by Levi-Montalcini as being a “*circular or ellipsoidal*, *perfectly geometrical ring around the explant*”[17]. Although an ellipse can theoretically be defined with the explant core at its center, the examples illustrated by Levi-Montalcini and colleagues [for example, Fig 3B in [14] as well as our results], almost always have the core nearer to one focus of an ellipse. Furthermore, the long trail of neurites often present on one side gives the explant the appearance of a teardrop or comet-shaped halo. Of the published images in the literature, some show mainly radially-symmetric outgrowth [9,10] but there are numerous examples of asymmetric halos as well, e.g., Fig 4B of [18], Fig 1B in [19], Fig 2 in [20], Fig 7 in [21], Fig 2A in [22], and Fig 1 in [23]. However, many published images show only a small portion of the explant and/or its halo so that it is not possible to determine whether asymmetric outgrowth occurred.

As in the original work involving co-cultures of neural tissue with non-neural tissue, preferential outgrowth from neural explants co-cultured with other tissue types has been documented [24–30]. Other studies have used orienting stimuli, such as a local source of a neurotrophic factor [9] or application of an electric field [31,32] to elicit asymmetric outgrowth. In most such studies, there is little information provided on the prevalence of asymmetry in control cultures.

Unlike other studies where asymmetric outgrowth was reported in the presence of other tissue or some other stimulus, the explants in the present study were cultured in nominally homogeneous conditions. Although not all explants showed asymmetry, neurite outgrowth from embryonic chick sympathetic ganglia was at least 15% asymmetric in 73% of the explants quantified [the mode is 35% neurite length asymmetry, approximately that shown in the example shown in Fig 3C]. Asymmetric outgrowth did not depend on the presence of exogenous NGF as long as other conditions permitted the establishment of a neurite halo, e.g., the use of Neurobasal medium [unpublished observations], although the presence of NGF may increase asymmetry, perhaps by promoting more rapid outgrowth. Asymmetry in neurite length and morphology did not always occur together [Fig 8] but when they did, the shorter neurites were inevitably those with more flattened club-like endings, possibly growth cones. It is also possible that some of the variability in the extent of asymmetry could be due to cell death or neurite degeneration that would not be visible with the methods used here.

The mechanism[s] underlying the asymmetry and co-orientation is uncertain. Detection of co-orientation depends on the presence of halo asymmetry. In fact, it was the co-orientation that first brought attention to the asymmetric outgrowth. However, whether a common mechanism accounts for both phenomena is not clear. Although individual neurons exhibited extensive neurite outgrowth under the same culture conditions, co-orientated growth was only observed in explants or re-aggregated cell clusters. This suggests that co-oriented outgrowth is an emergent property requiring a minimal number of cells analogous to what has been termed the “community effect” in other systems, such as the differentiation of muscle cells [33–39].

In light of the evidence that various stimuli can influence neurite outgrowth in culture, including diffusible growth factors, substrate-bound factors, and bioelectric phenomena, a number of hypotheses can be envisioned. However, we have no direct evidence to distinguish among these other than the fact that the orientation of outgrowth is not consistent from dish to dish in the same experiment, which argues against a global field effect, e.g., gravity. One could speculate on various mechanisms that might be involved in the development of asymmetry as neurites extend and interact, such as the role of protocadherins in mediating neurite interactions [40]. At this stage, however, we are confined to primarily qualitative observations that require future work to uncover possible mechanisms.

If the in vitro observations reported here are at least partly attributable to phenomena that occur during development, further studies of explants cultured under similar conditions could reveal relevant mechanisms also operating in vivo. It would also be informative to determine whether these phenomena occur in explants of other neural tissue as a function of developmental age, anatomical location or species. At the very least, the co-oriented asymmetric outgrowth reported here suggests that there are tissue-level mechanisms that serve to organize neural tissue in a way not previously reported. Additional experiments will be required to account for the mechanism underlying both the expression of asymmetry and the co-orientation of neuritic halos.
